# Effect of a WeChat-Based Intervention (Run4Love) on Depressive Symptoms Among People Living With HIV in China: Randomized Controlled Trial

**DOI:** 10.2196/16715

**Published:** 2020-02-11

**Authors:** Yan Guo, Y Alicia Hong, Weiping Cai, Linghua Li, Yuantao Hao, Jiaying Qiao, Zhimeng Xu, Hanxi Zhang, Chengbo Zeng, Cong Liu, Yiran Li, Mengting Zhu, Yu Zeng, Frank J Penedo

**Affiliations:** 1 Department of Epidemiology and Biostatistics School of Public Health Sun Yat-sen University Guangzhou China; 2 Sun Yat-sen Center for Migrant Health Policy Guangzhou China; 3 Sun Yat-sen Center for Global Health Guangzhou China; 4 Department of Health Administration and Policy College of Health and Human Services George Mason University Fairfax, VA United States; 5 Department of Infectious Diseases Guangzhou Number Eight People’s Hospital Guangzhou China; 6 National Center of AIDS/STD Control and Prevention Beijing China; 7 South Carolina SmartState Center of Healthcare Quality Arnold School of Public Health University of South Carolina Columbia, SC United States; 8 Department of Health Promotion, Education, and Behavior Arnold School of Public Health University of South Carolina Columbia, SC United States; 9 Department of Psychology University of Miami Coral Gables, FL United States; 10 Sylvester Comprehensive Cancer Center Miller School of Medicine University of Miami Miami, FL United States

**Keywords:** HIV, depression, mHealth, WeChat, randomized controlled trial

## Abstract

**Background:**

People living with HIV (PLWH) have high rates of depressive symptoms. However, only a few effective mental health interventions exist for this vulnerable population.

**Objective:**

The aim of this study was to assess the efficacy of a WeChat-based intervention, Run4Love, with a randomized controlled trial among 300 people living with HIV and depression (PLWHD) in China.

**Methods:**

We recruited PLWH from the HIV outpatient clinic in South China. Participants were screened based on the Center for Epidemiologic Studies-Depression (CES-D) scale. Those who scored 16 or higher were eligible to participate. A total of 300 eligible patients were enrolled. After obtaining informed consent from the participants, completion of a baseline survey, and collection of participants’ hair samples for measuring cortisol, the participants were randomly assigned to an intervention or a control group in a 1:1 ratio. The intervention group received the Run4Love program, delivered via the popular social media app WeChat. Cognitive behavioral stress management courses and weekly reminders of exercise were delivered in a multimedia format. Participants’ progress was monitored with timely and tailored feedback. The control group received usual care and a brochure on nutrition for PLWH. Data were collected at 3, 6, and 9 months. The primary outcome was depression, which was measured by a validated instrument.

**Results:**

Participants in the intervention and control groups were comparable at baseline; about 91.3% (139/150), 88.3% (132/150), and 86.7% (130/150) participants completed the 3-, 6-, and 9-month follow-ups, respectively. At the 3-month follow-up, a significant reduction in CES-D score was observed in the intervention group (from 23.9 to 17.7 vs from 24.3 to 23.8; mean difference=−5.77, 95% CI −7.82 to −3.71; *P*<.001; standard effect size *d*=0.66). The mean changes in CES-D score from baseline to the 6- and 9-month follow-ups between the two groups remained statistically significant. No adverse events were reported.

**Conclusions:**

The WeChat-based mobile health (mHealth) intervention Run4Love significantly reduced depressive symptoms among PLWHD, and the effect was sustained. An app-based mHealth intervention could provide a feasible therapeutic option for many PLWHD in resource-limited settings. Further research is needed to assess generalizability and cost-effectiveness of this intervention.

**Trial Registration:**

Chinese Clinical Trial Registry ChiCTR-IPR-17012606; http://www.chictr.org.cn/showproj.aspx?proj=21019 (Archived by WebCite at https://www.webcitation.org/78Bw2vouF)

## Introduction

People living with HIV (PLWH) are twice as likely to have depressive symptoms than the general population [1], and nearly 1 in 3 PLWH meet the criteria for depression [2]. Of the 36.7 million PLWH in the world, more than 12 million are people living with elevated depressive symptoms or people living with HIV and depression (PLWHD) [3]. The World Health Organization (WHO) recommends mental health services for all PLWH [4]. However, only a few effective mental health interventions exist for this vulnerable population, especially in middle- and low-income countries, where more than 90% of PLWH live [5]. In China, because of a shortage of mental health professionals, more than half (52%) of the people with mental disorders have never used mental health services [6]. Furthermore, because of a high level of HIV-related stigma, very few PLWHD have ever received any treatment or care for their depressive symptoms [7].

Widely accessible mobile tools offer a promising intervention delivery mode to serve a large number of PLWHD. In China, more than 95% of adults own a mobile phone and over 1 billion access WeChat, a popular mobile app, at least once a day [8]. However, existing mobile health (mHealth) interventions for PLWH were mostly feasibility studies with small samples and pre-post designs or those typically used phone calls or SMS with a focus on medication adherence [9-12]. Despite a growing interest in mHealth interventions among PLWH, especially their initial efficacy in improving medication adherence [12,13], few mHealth interventions exist for improving mental health outcomes of PLWHD. Data are further scarce from such studies based on a randomized controlled trial (RCT) [14].

We conducted an RCT (Chinese Clinical Trial Registry: ChiCTR-IPR-17012606) of Run4Love, a WeChat-based mHealth intervention aimed to reduce depressive symptoms among PLWHD, with 3-, 6-, and 9-month follow-ups. In the Run4Love study, we used an enhanced WeChat platform to deliver a culturally adapted, evidence-based cognitive behavioral stress management (CBSM) course and to promote regular physical activity in PLWHD [15]. We hypothesized that the intervention group would have greater improvement in the measures of depressive symptoms, quality of life (QOL), and other psychosocial outcomes, compared with the control group in usual care.

## Methods

### Study Design

The study was a parallel-group RCT. It was conducted in Guangzhou, China, from September 2017 to October 2018. Participants were randomized into two groups in a 1:1 ratio: a WeChat-based mHealth intervention group or a usual care waitlist control group. The study design was detailed in the Consolidated Standards Of Reporting Trials–eHealth checklist in the Multimedia Appendix 1. The study protocol was approved by the Institutional Review Board of Sun Yat-sen University.

### Participants

Participants were recruited from the outpatient clinic of the only hospital designated for HIV treatment in Guangzhou, the third largest city in China. The hospital treated over 14,000 PLWH. Patients in the waiting area were invited by the research staff to participate in the study. Patients first completed a brief screening questionnaire in a private space; those who met the eligibility criteria (see below) were provided with a pamphlet describing the Run4Love study and were then invited to join the study. Patients interested to participate were given further information about the study. After providing the written informed consent, eligible patients completed a baseline survey on a tablet and provided their hair samples.

The inclusion criteria were as follows: (1) being 18 years or older, (2) being HIV seropositive, (3) having elevated depressive symptoms (measured by the Center for Epidemiologic Studies-Depression Scale [CES-D] ≥16), (4) willing to provide hair samples, and (5) using WeChat.

The criteria for exclusion were as follows: (1) currently on psychiatric treatment, (2) unable to finish the screening or baseline survey, (3) unable to read or listen to the materials sent via WeChat (ie, short articles, audio, and posters), and (4) unable to engage in physical activities because of medical reasons.

### The Run4Love Intervention

The intervention protocol has been detailed elsewhere [15]. Briefly, participants in the intervention group received a 3-month Run4Love program, comprising two major components: the adapted CBSM course [16] and physical activity promotion. The adapted CBSM course included nine sessions and three review sessions on stress reduction management and coping skills such as muscle relaxation, breathing, and meditation, which was in a multimedia format and sent 3 to 5 times weekly. The articles were on average of 1300 words and took about 5 min to read; the audios took 5 to 10 min to listen to. The physical activity promotion program comprised goal setting and personalized feedback in addition to information on benefits of and guidance for regular exercise and healthy diet. The program was delivered via the enhanced WeChat platform with added functions of automated information sending, progress tracking on course completion and physical activity, and weekly personalized feedback. The most read articles were selected and sent to the participants weekly as a booster in the 3 months postintervention.

Participants in the waitlist control group received a brochure on nutrition in addition to usual care for HIV treatment. The design of the Run4Love intervention and the control condition was based on our previous research on PLWH and a pilot mHealth intervention [17-19].

### Randomization and Masking

Allocation to the treatment group was carried out by a computer-generated randomization list with a block size of 4, using SAS software version 9.4 (SAS Institute, Inc). By the nature of the trial design, neither the research staff nor the participants were blinded to the intervention.

### Quality Control and Participant Retention

We used multiple means for quality control and participant retention. The back end of the Run4Love account could track the course completion, which allowed us to send personalized feedback and reminders based on the participants’ progress. Participants in the intervention group also received up to 5 phone calls from the research staff at week 1 and month 1, 2, 5, and 8 after enrollment. The phone call in the first week was to confirm participation and ensure participants’ proper use of the Run4Love WeChat account. The other phone calls were to identify the barriers to intervention adherence, provide feedback, and remind the participants of regular medical checkups.

### Outcomes

All outcomes were measured at baseline before randomization and at follow-ups. The self-report psychosocial measures were collected at baseline, and at 3-, 6-, and 9-month follow-ups using electronic questionnaires on a tablet. The hair samples were collected at baseline and the 3-month follow-up.

#### Primary Outcome

The primary outcome was the change in depressive symptoms based on CES-D, Chinese version, measured at baseline, and at the 3-, 6-, and 9-month follow-ups. CES-D is a validated instrument for assessing depressive symptoms, and it has been used in various contexts and populations, including the Chinese PLWH [20]; it assesses participants’ depressive symptoms in the past week, with 20 items measuring 4 dimensions (ie, positive affect, depressed affect, interpersonal relationship, and somatic and retarded activity) [20]. The scores of CES-D range from 0 to 60, with CES-D scores ≥16 being considered as possible clinical depression and higher scores indicating more severe depressive symptoms [21].

#### Secondary Outcomes

Secondary outcomes included 3-, 6-, and 9-month changes in QOL, 9-item Patient Health Questionnaire (PHQ-9), self-efficacy, perceived stress, positive and negative coping, HIV-related stigma, and physical activity. All these outcomes were measured with surveys administered on a tablet. The last secondary outcome was chronic stress measured by cortisol in hair samples.

QOL was measured by the World Health Organization Quality of Life HIV short version (WHOQOL-HIV BREF) for PLWH, with 31 items assessing six domains (ie, physical, psychological, level of independence, social relationships, environment, and beliefs) [22]. The scores of WHOQOL-HIV BREF range from 24 to 120, with higher scores indicating better QOL. PHQ-9 is a 9-item validated instrument for major depressive disorder based on the *Diagnostic and Statistical Manual of Mental Disorders*, with 10 as a cutoff point for depression and a higher score indicating a higher level of depression [23-25]. Self-efficacy was measured by the 10-item General Self-Efficacy Scale (GSES), Chinese version (range 10-40, a higher score indicates a higher level of self-efficacy) [26]. A measure of stress was the 10-item Perceived Stress Scale (PSS), with a range of 0 to 40 and a higher score indicating more stress [27]. Coping was assessed by the Simplified Ways of Coping Questionnaire (SWCQ), Chinese version, with 12 items (score range 0-36) measuring positive coping and 8 items measuring negative coping (score range 0-24); higher scores indicate higher levels of positive or negative coping [28]. HIV-related stigma was assessed by 14 items derived from the HIV Stigma Scale measuring internalized and perceived stigma, with higher scores representing higher levels of stigma [29]. Physical activity was measured by the Chinese version of the Global Physical Activity Questionnaire (GPAQ), which is widely used in people with chronic diseases [30]. Metabolic equivalents (METs) calculated from GPAQ were used to measure the intensity of physical activities, with METs ≥600 indicating that individuals meet the minimum requirement of the WHO’s recommendation of weekly exercise intensity.

We also collected participants’ hair samples to test the cortisol content in the past month as a biomarker of chronic stress at baseline and the 3-month follow-up [15]. However, hair samples were not collected properly, resulting in insufficient weight for machine reading; therefore, cortisol data were not available.

#### Exploratory Outcomes

The outcome not prespecified in the protocol was change of proportion of clinical depression (Centre for Epidemiological Studies Depression >16) from baseline to 3, 6, and 9 months. We also assessed patient satisfaction for participating in the program.

### Statistical Analysis

The intention-to-treat principle was applied to all analyses [31]. Baseline characteristics were summarized as means and SDs for continuous measures and as numbers and percentages for categorical measures in each group. Baseline characteristics were compared between the groups using two-sample two-tailed *t* tests for continuous measures and using chi-square (χ^2^) tests for categorical measures. For between-group differences, 95% CIs were calculated for continuous measures. Analyses for changes in outcomes between baseline and each follow-up were performed using multiple imputations for the missing data [31]. The R package *mice* (R Foundation, version 3.4.2) was used to obtain 80 imputed data sets. Variables used for imputation included age, gender, marital status, sexual orientation, education, BMI, family monthly income, household registration, duration of HIV infection, and outcome values.

For the primary outcome, group differences in CES-D scores over the 9 months of the trial were estimated using a linear mixed-effect model (LMM) with repeated measures, adjusting for baseline CES-D score, time, and other baseline characteristics, including age, gender, BMI, education, sexual orientation, family monthly income, marital status, duration of HIV infection, and employment [32]. In addition, interactions between group and time were also examined in LMM. R package *nlme* (R Foundation, version 3.4.2) was used to conduct the LMM analysis.

Similar analyses were repeated for secondary outcomes. In post hoc exploratory analyses, the effect of the intervention on the 3-month change in the CES-D score was evaluated using tests for interaction to determine statistical significance in subsets of participants grouped by baseline characteristics.

Analyses were performed using R version 3.4.2, and a two-sided *P*<.05 was considered as statistically significant. As multiple secondary outcomes were compared, a two-sided *P*<.005 was considered statistically significant for secondary outcomes.

## Results

### Sample Characteristics

**Figure 1:**
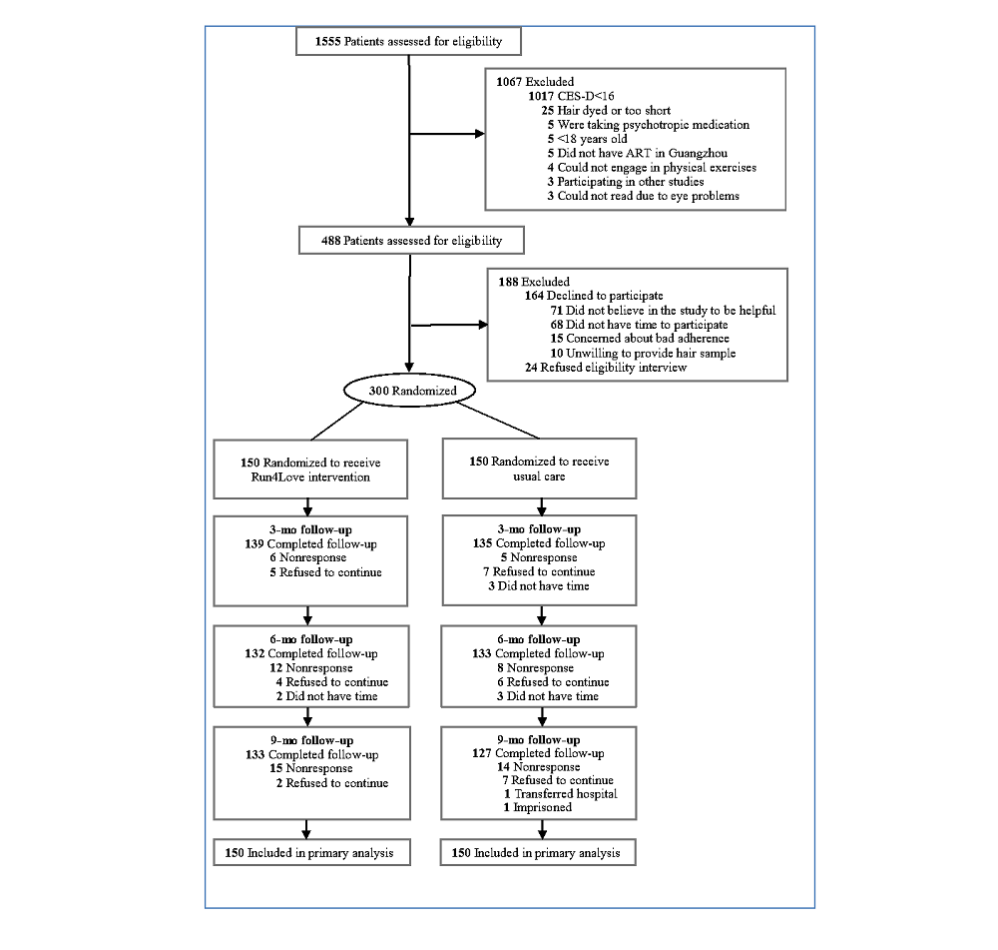
Flowchart of participant screening and recruitment.

Figure 1 summarizes the flow of participants through the study. Of the 1555 patients who were screened and provided information about the depressive symptoms measured by the CES-D scale, 1067 patients were excluded as their scores were lower than 16, and 488 patients were eligible for further interview. In the end, a total of 300 patients met the eligibility criteria and completed the baseline assessment before being randomized into the trial with 150 patients in each group (Figure 1). The mean (SD) age of the participants was 28.3 (5.8) years. Of the 300 participants, 277 (92.3%) were men and 245 (81.7%) were homosexual or bisexual or uncertain of their sexual orientation. The follow-up rates were 91.3% (92.7% in the intervention group and 90.0% in the control group), 88.3% (88.0% in the intervention group and 88.7% in the control group), and 86.7% (88.7% in the intervention group and 84.7% in the control group) at 3, 6, and 9 months, respectively. Moreover, participants in the intervention group completed, on average, 55% of the CBSM coursework at 3 months.

Except for the fact that the intervention group had a slightly higher proportion of participants with homosexual or bisexual or uncertain sexual orientation, the baseline characteristics were balanced between the two groups (Table 1). Those lost to follow-up were older than those who completed the trial; however, other characteristics were balanced between nonrespondents and respondents (see Multimedia Appendix 2).

**Table 1:** Baseline characteristics of participants in the intervention and control groups.

Baseline characteristics	Run4Love intervention (N=150)	Usual care (N=150)	*P* value
Age (years), mean (SD)	28.0 (5.8)	28.6 (5.9)	.39
Male, n (%)	142 (94.7)	135 (90.0)	.13
Body mass index^a^, mean (SD)	20.5 (2.5)	20.1 (2.4)	.19
Educational level >high school, n (%)	98 (65.3)	84 (56.0)	.10
Homosexual/bisexual/uncertain, n (%)	130 (86.7)	115 (76.7)	.03
Married, n (%)	18 (12.0)	20 (13.3)	.73
Employed, n (%)	123 (82.0)	128 (85.3)	.17
Family monthly income ≥7000 (yuan), n (%)	68 (45.3)	56 (37.3)	.16
Duration of HIV infection, mean (SD)	2.4 (2.3)	2.3 (2.3)	.62
Center for Epidemiological Studies Depression Scale^b^, mean (SD)	23.9 (6.4)	24.3 (6.9)	.68
Depression severity (9-item Patient Health Questionnaire^c^), mean (SD)	10.2 (4.5)	10.7 (5.1)	.31
Physical activity (metabolic equivalents ≥600), n (%)	65.0 (43.3)	65.0 (43.3)	.00
Quality of life^d^, mean (SD)	77.4 (9.0)	76.6 (9.4)	.41
Self-efficacy (General Self-Efficacy Scale^e^), mean (SD)	24.4 (5.2)	23.3 (5.6)	.08
Perceived stress (Perceived Stress Scale^f^), mean (SD)	20.0 (4.4)	20.7 (4.4)	.15
HIV Stigma Scale^g^, mean (SD)	37.1 (7.7)	38.0 (7.5)	.31
Simplified Ways of Coping Questionnaire positive coping^h^, mean (SD)	18.4 (5.5)	18.3 (6.2)	.92
Simplified Ways of Coping Questionnaire negative coping^i^, mean (SD)	11.8 (3.9)	11.8 (3.9)	.94
CD4^j^, mean (SD)	431 (192)	424 (195)	.74

^a^Calculated as weight in kilograms divided by height in meters squared.

^b^The Center for Epidemiological Studies Depression Scale score range 0 to 60; higher scores indicate worse depression.

^c^9-item Patient Health Questionnaire score range 0 to 27; higher scores indicate worse depression.

^d^HIV-related quality of life score range 24 to 120; a higher score indicates a better outcome.

^e^General Self-efficacy Scale score range 10 to 40; a higher score indicates a better outcome.

^f^Perceived Stress Scale score range 0 to 40; a higher score indicates a worse outcome.

^g^HIV Stigma Scale score range 14 to 56; a higher score indicates a worse outcome.

^h^Simplified Ways of Coping Questionnaire positive coping domain score range 0 to 36; a higher score indicates a better outcome.

^i^Simplified Ways of Coping Questionnaire negative coping domain score range 0 to 24; a higher score indicates a worse outcome.

^j^A higher score indicates a better outcome.

### Primary Outcome

**Figure 2:**
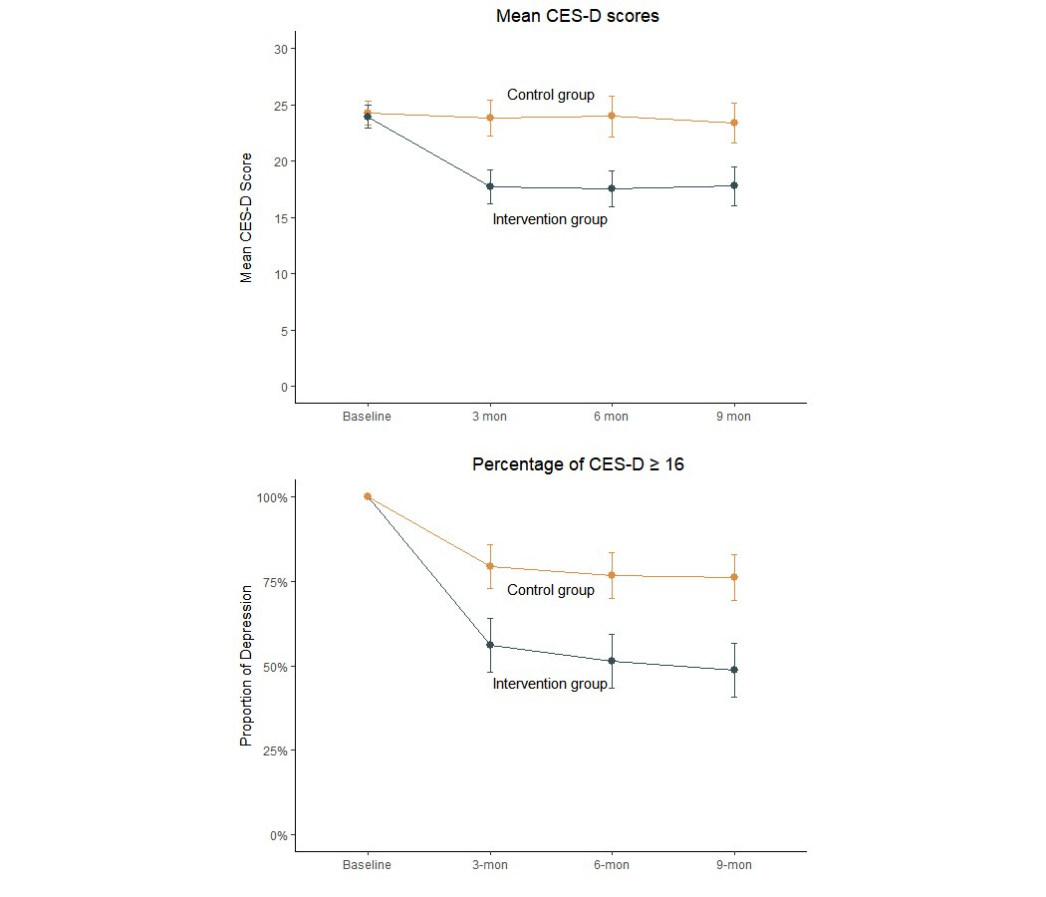
Depression severity and percentage change over time for the intervention group vs the control group.

The results of changes in depression are summarized in Table 2. At the 3-month follow-up, participants in the intervention group had significantly reduced depression severity (CES-D) compared with the control group (from 23.9 to 17.7 vs from 24.3 to 23.8; mean difference=−5.77, 95% CI −7.82 to −3.71; *P*<.001), with a standard effect size (Cohen *d*) of 0.66 in favor of the Run4Love intervention (Multimedia Appendix 2). At the 6- and 9-month follow-ups, between-group differences in the CES-D score remained statistically significant (6-month follow-up: −6.08, 95% CI −8.33 to −3.83; *P*<.001; Cohen *d*=0.63; and 9-month follow-up: −5.30, 95% CI −7.77 to −2.83; *P*<.001; Cohen *d*=0.51). LMM indicated significant interactions between the groups and at each follow-up time (at 3, 6, and 9 months), with statistically significant between-group differences in the CES-D score for mean change from baseline, controlling for baseline characteristics (*P*<.001; Table 2). Changes over time are presented in Figure 2. The results were not substantially different from data gathered before multiple imputations of missing data (Multimedia Appendix 2).

**Table 2:** Effects of the intervention on primary outcome (Centre for Epidemiological Studies Depression score).

Follow-up time	Within-group changes, mean (95% CI)^a,b^		Between-group difference for mean change from baseline (95% CI)	*P* value	Linear mixed-effect model results, *P* value^c^		
	Run4Love intervention group (N=150)	Usual care group (N=150)			Group	Time	Group×time
Baseline^d^	—^e^	—	—	—	.85	—	—
3 months^f^	–6.21 (–7.66 to –4.76)	–0.44 (–1.92 to 1.03)	–5.77 (–7.82 to –3.71)	<.001	—	.62	<.001
6 months	–6.37 (–7.96 to –4.79)	–0.29 (–1.93 to 1.34)	–6.08 (–8.33 to –3.83)	<.001	—	.76	<.001
9 months	–6.17 (–7.99 to –4.35)	–0.87 (–2.54 to 0.81)	–5.30 (–7.77 to –2.83)	<.001	—	.40	<.001

^a^Indicates mean change between baseline and follow-up.

^b^Higher scores indicate greater depression.

^c^Adjusted for age, gender, body mass index, education, sexual orientation, family monthly income, marital status, duration of HIV infection, and employment.

^d^H0, the risk difference equals to zero.

^e^Not applicable.

^f^Primary end point.

### Secondary Outcomes

At the 3-month follow-up, participants in the Run4Love intervention group, when compared with the control group, had significantly improved QOL (WHOQOL-HIV BREF: from 77.4 to 82.6 vs 76.6 to 77.0; mean difference=4.79, 95% CI 2.72 to 6.87; *P*<.001), self-efficacy (GSES: from 24.4 to 26.6 vs from 23.3 to 23.4; mean difference=2.16, 95% CI 0.92 to 3.40; *P*<.001), and SWCQ positive coping (from 18.4 to 20.7 vs from 18.3 to 17.8; mean difference=2.91, 95% CI 1.39 to 4.43; *P*<.001; Table 3, for more details, see Multimedia Appendix 2). In comparison with the control group, participants in the intervention group also had significantly reduced perceived stress (PSS: from 20.0 to 15.7 vs from 20.7 to 18.9; mean difference=−2.45, 95% CI −3.63 to −1.27; *P*<.001) and depression severity (PHQ-9: from 10.2 to 6.8 vs from 10.7 to 8.9; mean difference=−1.56, 95% CI −2.63 to −0.50; *P*=.004). There were no significant between-group differences in changes in SWCQ negative coping, HIV-related stigma (HIV Stigma Scale), physical activity (METs; *P*>.005; Table 3; for more details, see Multimedia Appendix 2).

At the 6- and 9-month follow-ups, the between-group differences remained statistically significant for QOL (6-month follow-up: 6.6, 95% CI 4.24 to 8.87; *P*<.001 and 9-month follow-up: 5.84, 95% CI 2.76 to 8.31; *P*<.001) and SWCQ positive coping (6-month follow-up: 3.41, 95% CI 1.80 to 5.02; *P*<.001 and 9-month follow-up: 2.53, 95% CI 0.85 to 4.21; *P*=.003) but not for self-efficacy (GSES at the 6-month follow-up: 1.86, 95% CI 0.50 to 3.22; *P*=.007 and GSES at the 9-month follow-up: 1.55, 95% CI 0.19 to 2.91; *P*=.03). At 6 months, between-group differences remained statistically significant for perceived stress (PSS: −1.88, 95% CI −3.10 to −0.67; *P*=.003) and depression severity (PHQ-9: −2.01, 95% CI −3.20 to −0.83; *P*<.001) but not for the 9-month follow-up (perceived stress: −1.79, 95% CI −3.06 to −0.53, *P*=.006; PHQ-9: −1.17, 95% CI −2.46 to 0.13; *P*=.08). At 9 months, participants in the intervention group had significantly reduced HIV-related stigma compared with the control group (HIV Stigma Scale between-group difference: −2.87, 95% CI −4.71 to −1.03; *P*=.002), which did not occur at 3 months (between-group difference: −2.29, 95% CI −3.93 to −0.65; *P*=.006) or 6 months (between-group difference: −2.05, 95% CI −3.83 to −0.28; *P*=.02). There were no statistically significant between-group differences in change in SWCQ negative coping and physical activity (METs) from baseline to the 3-, 6-, and 9-month follow-ups (Table 3; for more details, see Multimedia Appendix 2). The results from LMM did not substantially change after controlling for baseline characteristics (Multimedia Appendix 2).

As reported in the Methods section, hair samples were not properly collected; therefore, we did not have cortisol data. No adverse events were reported.

**Table 3:** Effects of the intervention on secondary outcomes.

Follow-up time		Within-group changes, mean (95% CI)^a^			Between-group difference for mean change from baseline (95% CI)		*P* value
		Run4Love intervention group (N=150)	Usual care group (N=150)				
**Quality of life^b^**							**<.001**
	3 months	5.16 (3.55 to 6.76)	0.36 (−0.96 to 1.68)	4.79 (2.72 to 6.87)			
	6 months	6.26 (4.50 to 8.01)	−0.34 (−1.87 to 1.19)	6.6 (4.27 to 8.92)			
	9 months	5.91 (3.89 to 7.93)	0.07 (−1.66 to 1.8.0)	5.84 (3.18 to 8.51)			
**Perceived stress (Perceived Stress Scale^c^)**							
	3 months	−4.23 (−5.08 to −3.38)	−1.78 (−2.61 to −0.95)	−2.45 (−3.63 to −1.27)		<.001	
	6 months	−3.35 (−4.23 to −2.46)	−1.46 (−2.32 to −0.60)	−1.88 (−3.10 to −0.67)		.003	
	9 months	−3.84 (−4.74 to −2.93)	−2.04 (−2.94 to −1.14)	−1.79 (−3.06 to −0.53)		.006	
**Simplified Ways of Coping Questionnaire positive coping^b^**							
	3 months	2.35 (1.18 to 3.52)	−0.56 (−1.53 to 0.41)	2.91 (1.39 to 4.43)		<.001	
	6 months	2.48 (1.30 to 3.65)	−0.93 (−2.04 to 0.17)	3.41 (1.80 to 5.02)		<.001	
	9 months	2.44 (1.16 to 3.72)	−0.09 (−1.22 to 1.03)	2.53 (0.85 to 4.21)		.003	
**Simplified Ways of Coping Questionnaire negative coping^c^**							
	3 months	−0.67 (−1.40 to 0.06)	−0.29 (−0.93 to 0.35)	−0.38 (−1.34 to 0.58)		.44	
	6 months	−0.45 (−1.17 to 0.27)	−0.44 (−1.14 to 0.26)	−0.01 (−1.00 to 0.99)		.99	
	9 months	−0.06 (−0.8 to 0.68)	0.05 (−0.72 to 0.82)	−0.11 (−1.18 to 0.96)		.84	
**Physical activity (metabolic equivalents^b^)**							
	3 months	−155 (−1301 to 990)	1743 (−370 to 3856)	−1898 (−4285 to 489)		.12	
	6 months	1193 (−775 to 3161)	1296 (−525 to 3116)	−103 (−2769 to 2564)		.94	
	9 months	1482 (−235 to 3199)	1792 (76 to 3508)	−310 (−2713 to 2094)		.80	
**Self-efficacy (General Self-Efficacy Scale^b^)**							
	3 months	2.24 (1.33 to 3.15)	0.08 (−0.77 to 0.94)	2.16 (0.92 to 3.40)		<.001	
	6 months	2.06 (1.09 to 3.03)	0.20 (−0.76 to 1.15)	1.86 (0.50 to 3.22)		.007	
	9 months	2.01 (1.08 to 2.95)	0.46 (−0.55 to 1.47)	1.55 (0.19 to 2.91)		.03	
**HIV Stigma Scale^c^**							
	3 months	−2.85 (−4.09 to −1.61)	−0.56 (−1.65 to 0.53)	−2.29 (−3.93 to −0.65)		.006	
	6 months	−2.88 (−4.10 to −1.66)	−0.82 (−2.14 to 0.49)	−2.05 (−3.83 to −0.28)		.02	
	9 months	−3.15 (−4.43 to −1.86)	−0.28 (−1.60 to 1.04)	−2.87 (−4.71 to −1.03)		.002	
**Depression severity (9-item Patient Health Questionnaire^c^)**							
	3 months	−3.38 (−4.13 to −2.62)	−1.81 (−2.58 to −1.05)	−1.56 (−2.63 to −0.50)		.004	
	6 months	−1.68 (−2.54 to −0.81)	0.34 (−0.49 to 1.16)	−2.01 (−3.20 to −0.83)		<.001	
	9 months	−1.01 (−1.99 to −0.03)	0.16 (−0.70 to 1.02)	−1.17 (−2.46 to 0.13)		.08	

^a^Within-group changes are mean changes.

^b^A higher score indicates a better outcome.

^c^A higher score indicates a worse outcome.

### Exploratory Analyses

In the post hoc exploratory analyses, the proportion reduction in depression measured by CES-D ≥16 was greater in the intervention group than in the control group (Table 2). The between-group differences in the proportion reduction in depression (CES-D ≥16) were 23.3%, 25.3%, and 27.3% at 3, 6, and 9 months, respectively, in favor of the Run4Love intervention (3-month proportion reduction: 44.0% vs 20.7%; *P*<.001; 6-month proportion reduction: 48.7% vs 23.3%; *P*<.001; 9-month proportion reduction: 51.3% vs 24.0%; *P*<.001; Table 4). In addition, patients in the intervention and control groups reported high levels of satisfaction (92%-97%) at all three assessments, and there were no significant differences between the groups (see Multimedia Appendix 2 for details).

**Table 4:** Effects of the intervention on exploratory outcomes.

Follow-up time for CES-D^a^ ≥16	Run4Love intervention group (N=150)		Usual care group (N=150)		Between-group difference in percentage points (95% CI)	*P* value
	n (%)	95% CI	n (%)	95% CI		
3 months	84 (56.0)	48.1 to 63.9	119 (79.3)	72.9 to 85.8	−23.3 (−33.6 to −13.1)	<.001
6 months	77 (51.3)	43.3 to 59.3	115 (76.7)	69.9 to 83.4	−25.3 (−35.8 to −14.9)	<.001
9 months	73 (48.7)	40.7 to 56.7	114 (76.0)	69.2 to 82.8	−27.3 (−37.9 to −16.8)	<.001

^a^CES-D: Centre for Epidemiological Studies Depression.

### Subgroup Analyses

Except for age and marital status, there were no statistically significant interactions in the post hoc exploratory analyses, including gender, education, sexual orientation, family income, duration of HIV infection, or baseline CES-D score (Table 5). Those who were younger and not married had statistically significant improvement (between-group differences) in the CES-D score than those who were older or married (Table 5).

**Table 5:** Change in depression in study groups by participant characteristics.

Variables		Run4Love intervention (N=150)				Usual care (N=150)					Mean between-group difference for change from baseline (95% CI)^c^		*P* value^d^		*P* value for interaction
		N		Within-Group 3-month change in CES-D from baseline, mean (95% CI)^a^		N		Within-Group 3-month change in CES-D from baseline, mean (95% CI)^b^							
**Age**														.**02**	
	<27 years		68		−7.29 (−9.31 to −5.27)		65		1.28 (−0.59 to 3.15)		−8.57 (−11.30 to −5.83)		<.001		
	≥27 years		82		−5.31 (−7.39 to −3.24)		85		−1.76 (−3.92 to 0.40)		−3.55 (−6.51 to −0.60)		.02		
**Gender**														**.89**	
	Male		142		−6.28 (−7.75 to −4.81)		135		−0.52 (−2.11 to 1.06)		−5.76 (−7.90 to −3.62)		<.001		
	Female		8		−4.88 (−15.33 to 5.58)		15		0.31 (−4.09 to 4.70)		−5.18 (−13.65 to 3.28)		.22		
**BMI**														**.58**	
	<18.5 kg/m^2^		34		−5.40 (−9.25 to −1.54)		37		1.48 (−1.54 to 4.49)		−6.88 (−11.62 to −2.14)		.005		
	≥18.5 kg/m^2^		116		−6.45 (−7.98 to −4.91)		113		−1.07 (−2.78 to 0.64)		−5.38 (−7.64 to −3.11)		<.001		
**Education**														**.05**	
	≤high school		52		−4.27 (−6.44 to −2.10)		66		−1.12 (−3.56 to 1.32)		−3.15 (−6.46 to 0.16)		.06		
	>high school		98		−7.24 (−9.12 to −5.35)		84		0.09 (−1.76 to 1.94)		−7.33 (−9.96 to −4.69)		<.001		
**Sexual orientation**														**.27**	
	Homosexual		130		−6.37 (−7.93 to −4.81)		115		−0.01 (−1.71 to 1.69)		−6.36 (−8.64 to −4.08)		<.001		
	Heterosexual		20		−5.14 (−9.46 to −0.82)		35		−1.86 (−4.95 to 1.24)		−3.29 (−8.31 to 1.73)		.19		
**Marital status**														**.006**	
	Married		18		−3.78 (−8.08 to 0.52)		20		−5.41 (−10.43 to −0.39)		1.63 (−4.77 to 8.04)		.61		
	Unmarried		132		−6.54 (−8.09 to −4.99)		130		0.32 (−1.19 to 1.83)		−6.86 (−9.01 to −4.71)		<.001		
**Family monthly income**														**.45**	
	≥7000 yuan		82		−6.22 (−8.12 to −4.32)		94		0.14 (−1.69 to 1.97)		−6.36 (−8.98 to −3.75)		<.001		
	>7000 yuan		68		−6.19 (−8.45 to −3.93)		56		−1.42 (−3.98 to 1.15)		−4.77 (−8.12 to −1.42)		.006		
**Employed**														**.17**	
	Employed		123		−5.68 (−7.29 to −4.08)		128		−0.63 (−2.25 to 0.99)		−5.05 (−7.32 to −2.79)		<.001		
	Unemployed		27		−8.59 (−12.09 to −5.09)		22		0.66 (−3.22 to 4.54)		−9.25 (−14.33 to −4.18)		<.001		
**Duration of HIV infection**														**.99**	
	≤1 year		54		−6.62 (−9.15 to −4.10)		56		−0.86 (−3.48 to 1.76)		−5.77 (−9.36 to −2.17)		.002		
	>1 year		96		−5.97 (−7.77 to −4.18)		94		−0.19 (−2.00 to 1.61)		−5.78 (−8.30 to −3.26)		<.001		
**Center for Epidemiologic Studies-Depression score**														**.83**	
	≤Baseline mean		91		−5.14 (−6.94 to −3.33)		93		0.77 (−1.02 to 2.56)		−5.91 (−8.42 to −3.39)		<.001		
	>Baseline mean		59		−7.86 (−10.30 to −5.43)		57		−2.42 (−4.96 to 0.12)		−5.44 (−8.91 to −1.97)		.002		

^a^Overall −6.21 (−7.66 to −4.76).

^b^Overall −0.44 (−1.92 to 1.03).

^c^Overall −5.77 (−7.82 to −3.71).

^d^Overall *P* value <.001.

## Discussion

### Overview

The WeChat-based mHealth intervention Run4Love significantly reduced depression severity measured by CES-D by 5.77 points at 3 months compared with usual care, and the improvement sustained at 6- and 9-month follow-ups, with a medium-to-large effect size of 0.66 at 3 months [33]. Between-group differences in depression (CES-D ≥16) proportion reduction were consistently more than 20% at the 3-, 6-, and 9-month follow-ups, in favor of the intervention group. The intervention also improved QOL, self-efficacy, SWCQ positive coping, reduced perceived stress, and depression severity (PHQ-9), compared with the control group at 3 months. The improvements in secondary outcomes such as QOL and SWCQ positive coping remained significant at the 6- and/or 9-month follow-ups. In addition to good efficacy, the Run4Love intervention demonstrated good feasibility as all participants reported a high level of satisfaction.

### Data Interpretation

The good effect sizes in the primary outcome and most secondary outcomes could be attributed to the following reasons. First, the study design was informed by extensive previous work, including a pilot mHealth intervention among PLWH [17-19]. Second, we culturally adopted the evidence-based CBSM, which had proven effect on stress management and positive coping [21]. Third, the Run4Love intervention was built on the enhanced WeChat platform, with functions of automatic distribution of multimedia programs, tracking of CBSM completion and physical activity, and personalized feedback and incentives. Fourth, we built our Run4Love mHealth intervention on a popular social media platform WeChat, used daily by most participants; it is easy for participants to join and use it continuously. Fifth, we established trust with participants at recruitment and continuously engaged them via tailored reminders, feedback, and incentives. Finally, the electronic questionnaire used for data collection ensured minimum missing values [34].

We also noted that older participants were also more likely to drop out of the study despite the high retention rate, suggesting a possible digital divide [34]. Our subgroup analyses showed that younger and unmarried participants benefited more from the intervention than their counterparts. Further research is required on the effective strategies to deliver mental health services to older PLWHD.

The literature documents that elevated depressive symptoms experienced by the PLWH are associated with the deteriorated immune system, worsened disease progression, poor QOL, increased risky behaviors, and increased mortality [35,36]. CBSM has been effective in improving mental health outcomes in PLWH [37]. However, the effects of these interventions delivered via mobile tools are understudied. A recent study by Schnall et al [10] randomized 80 low-income PLWHD who experienced at least two out of the 13 prespecified symptoms in the week before the mHealth intervention of “mobile Video Information Provider” with self-care strategies or a control group. At 12 weeks, participants in the intervention group showed improvements in 5 symptoms, including depression. However, this was a small-scale feasibility study conducted in New York City without follow-ups to examine long-term effects. To the best of our knowledge, the Run4Love intervention is one of the first RCTs of mobile app–delivered mHealth interventions among PLWHD with long-term and multiple follow-ups. It is also among the first efforts that adapted CBSM to a social media app and delivered it via a social media app to PLWHD. The findings of this study suggest that mHealth interventions could provide a feasible therapeutic option for many PLWHD in resource-poor settings where mental health services are limited but smartphones are widely accessible.

### Limitations

This RCT has several major limitations. First, most participants were young men who have sex with men recruited from a large hospital in South China. The findings from this study might not be generalizable to other PLWH, especially older PLWH, those living in rural areas, or those not infected through homosexual transmission. Second, intervention contamination was possible, and some participants might have shared the Run4Love information via their private WeChat accounts. However, the effect of such contamination was limited and, if any, only might have diluted the observed effect. Third, the intervention completion rates were suboptimal, but these were comparable with other internet-based cognitive behavioral therapy interventions [38]. With improved completion rates, intervention effects might be more pronounced. Fourth, the research staff was not blind to group allocation; however, the use of an electronic questionnaire on a tablet may limit the bias introduced by assessors. Finally, the improper collection of hair samples led to insufficient weight of hair and invalid readings. We therefore did not have cortisol data, which were planned for measuring chronic stress in our study protocol. More rigorous training and laboratory procedures are needed to ensure quality data collection. Future studies also need to include a robust biomarker to measure changes in mental health outcomes.

### Conclusions

The WeChat-based mHealth intervention Run4Love effectively reduced depressive symptoms in PLWHD, and the effect was sustainable at the 9-month follow-up. QOL and other psychosocial measures were also significantly improved at follow-ups. This RCT suggested that mHealth interventions to deliver mental health services to PLWH were feasible and effective, even in resource-limited settings, such as China. Further research is needed to assess the generalizability, biomarkers, and cost-effectiveness of such interventions.

## Appendix

Multimedia Appendix 1 CONSORT-EHEALTH checklist (V 1.6.1).

Multimedia Appendix 2 Supplementary tables.

## Abbreviations

CBSM: cognitive behavioral stress management
CES-D: Center for Epidemiologic Studies-Depression
GPAQ: Global Physical Activity Questionnaire
GSES: General Self-Efficacy Scale
LMM: linear mixed-effects model
METs: metabolic equivalents
mHealth: mobile health
PHQ-9: 9-item Patient Health Questionnaire
PLWH: people living with HIV
PLWHD: people living with HIV and depression
PSS: Perceived Stress Scale
QOL: quality of life
RCT: randomized controlled trial
SWCQ: Simplified Ways of Coping Questionnaire
WHO: World Health Organization
WHOQOL-HIV BREF: World Health Organization Quality of Life–HIV, short version
