# Supplementary material for: Effect of a WeChat-Based Intervention (Run4Love) on Depressive Symptoms Among People Living With HIV in China: Randomized Controlled Trial
Source: J Med Internet Res. 2020 Feb 11;22(2):e16715. doi: 10.2196/16715 (PMC7058168; doi:10.2196/16715)
Supplement: Multimedia Appendix 2 [file jmir_v22i2e16715_app2.docx]

**Supplementary Tables and Figures:**

eTable 1. Effects of Intervention on Primary and Secondary Outcomes (Full Results)

eTable 2. Effects of Intervention on Primary and Secondary Outcomes before Missing Data Imputation

eTable 3. Linear Mixed-Effects Model Results of the Primary Outcome and Secondary Outcomes

eTable 4. Effects of Intervention on Depression severity PHQ-9 ≥10

eTable 5. Generalized Estimating Equation Analysis of Run4Love Effects on Percentage of PHQ-9 ≥ 10

eFigure 1. Secondary Outcomes Over Time for The Run4Love Intervention vs Control Groups

eTable 6. Comparison of Participants between Who Were Lost Before 3-month Evaluation and Completed 3-month Outcome Evaluation

eTable 7. Comparison of Participants between Who Were Lost Before 6-month Evaluation and Completed 6-month Outcome Evaluation

eTable 8. Comparison of Participants between Who Were Lost Before 9-month Evaluation and Completed 9-month Outcome Evaluation

eTable 9. Effect Size of Intervention on Primary and Secondary Outcomes

eTable 10. Results of Patient Satisfaction between the Intervention and Control Groups

| **eTable 1 Effects of Intervention on Primary and Secondary Outcomes (Full Results)** | | | | | | | | | | | |
| --- | --- | --- | --- | --- | --- | --- | --- | --- | --- | --- | --- |
| **Follow-up time** | **Run4Love Intervention Group (*N*=150)** | | | | |  | **Usual Care Group (*N*=150)** | | | **Between-Group Difference for Mean Change From Baseline (95% *CI*)** | ***P* value** |
|  | **Baseline, Mean (*SD*)** | **Follow-up, Mean (*SD*)** | | **Within-Group Changes, Mean (95% *CI*)** ^c^ | |  | **Baseline, Mean (*SD*)** | **Follow-up, Mean (*SD*)** | **Within-Group Changes, Mean (95% *CI*)** ^c^ |  |  |
| CES-D^a^ |  |  | |  | |  |  |  |  |  |  |
| 3-mon-follow-up | 23.9 (6.4) | 17.7 (9.5) | | -6.21 (-7.66 to -4.76) | |  | 24.3 (6.9) | 23.8 (10.0) | -0.44 (-1.92 to 1.03) | -5.77 (-7.82 to -3.71) | <0.001 |
| 6-mon-follow-up | 23.9 (6.4) | 17.6 (10.0) | | -6.37 (-7.96 to -4.79) | |  | 24.3 (6.9) | 24.0 (11.2) | -0.29 (-1.93 to 1.34) | -6.08 (-8.33 to -3.83) | <0.001 |
| 9-mon-follow-up | 23.9 (6.4) | 17.8 (10.6) | | -6.17 (-7.99 to -4.35) | |  | 24.3 (6.9) | 23.4 (11.2) | -0.87 (-2.54 to 0.81) | -5.30 (-7.77 to -2.83) | <0.001 |
| QOL^b^ |  |  | |  | |  |  |  |  |  |  |
| 3-mon-follow-up | 77.4 (9.0) | 82.6 (12.0) | | 5.16 (3.55 to 6.76) | |  | 76.6 (9.4) | 77.0 (11.3) | 0.36 (-0.96 to 1.68) | 4.79 (2.72 to 6.87) | <0.001 |
| 6-mon-follow-up | 77.4 (9.0) | 83.7 (12.8) | | 6.26 (4.50 to 8.01) | |  | 76.6 (9.4) | 76.3 (12.7) | -0.34 (-1.87 to 1.19) | 6.6 (4.27 to 8.92) | <0.001 |
| 9-mon-follow-up | 77.4 (9.0) | 83.3 (13.0) | | 5.91 (3.89 to 7.93) | |  | 76.6 (9.4) | 76.7 (13.1) | 0.07 (-1.66 to 1.8.0) | 5.84 (3.18 to 8.51) | <0.001 |
| Perceived stress PSS^a^ |  |  | |  | |  |  |  |  |  |  |
| 3-mon-follow-up | 20.0 (4.4) | 15.7 (5.7) | | -4.23 (-5.08 to -3.38) | |  | 20.7 (4.4) | 18.9 (5.5) | -1.78 (-2.61 to -0.95) | -2.45 (-3.63 to -1.27) | <0.001 |
| 6-mon-follow-up | 20.0 (4.4) | 16.6 (5.8) | | -3.35 (-4.23 to -2.46) | |  | 20.7 (4.4) | 19.2 (6.0) | -1.46 (-2.32 to -0.60) | -1.88 (-3.10 to -0.67) | 0.003 |
| 9-mon-follow-up | 20.0 (4.4) | 16.1 (6.0) | | -3.84 (-4.74 to -2.93) | |  | 20.7 (4.4) | 18.7 (5.9) | -2.04 (-2.94 to -1.14) | -1.79 (-3.06 to -0.53) | 0.006 |
| SWCQ positive coping^b^ |  |  | |  | |  |  |  |  |  |  |
| 3-mon-follow-up | 18.4 (5.5) | 20.7 (7.3) | | 2.35 (1.18 to 3.52) | |  | 18.3 (6.2) | 17.8 (6.0) | -0.56 (-1.53 to 0.41) | 2.91 (1.39 to 4.43) | <0.001 |
| 6-mon-follow-up | 18.4 (5.5) | 20.9 (7.4) | | 2.48 (1.30 to 3.65) | |  | 18.3 (6.2) | 17.4 (6.6) | -0.93 (-2.04 to 0.17) | 3.41 (1.80 to 5.02) | <0.001 |
| 9-mon-follow-up | 18.4 (5.5) | 20.8 (7.7) | | 2.44 (1.16 to 3.72) | |  | 18.3 (6.2) | 18.2 (6.4) | -0.09 (-1.22 to 1.03) | 2.53 (0.85 to 4.21) | 0.003 |
| SWCQ negative coping^b^ |  |  | |  | |  |  |  |  |  |  |
| 3-mon-follow-up | 11.8 (3.8) | 11.1 (4.2) | | -0.67 (-1.40 to 0.06) | |  | 11.7 (3.9) | 11.5 (3.7) | -0.29 (-0.93 to 0.35) | -0.38 (-1.34 to 0.58) | 0.44 |
| 6-mon-follow-up | 11.8 (3.8) | 11.3 (4.4) | | -0.45 (-1.17 to 0.27) | |  | 11.7 (3.9) | 11.3 (4.1) | -0.44 (-1.14 to 0.26) | -0.01 (-1.00 to 0.99) | 0.99 |
| 9-mon-follow-up | 11.8 (3.8) | 11.7 (4.1) | | -0.06 (-0.8 to 0.68) | |  | 11.7 (3.9) | 11.8 (4.1) | 0.05 (-0.72 to 0.82) | -0.11 (-1.18 to 0.96) | 0.84 |
| Physical activity METs^b^ |  |  | |  | |  |  |  |  |  |  |
| 3-mon-follow-up | 3225 (6189) | 3070 (6036.6) | | -155 (-1301 to 990) | |  | 2675 (6064) | 4418 (15755) | 1743 (-370 to 3856) | -1898 (-4285 to 489) | 0.12 |
| 6-mon-follow-up | 3225 (6189) | 4418 (11870) | | 1193 (-775 to 3161) | |  | 2675 (6064) | 3970 (9090) | 1296 (-525 to 3116) | -103 (-2769 to 2564) | 0.94 |
| 9-mon-follow-up | 3225 (6189) | 4707 (10136) | | 1482 (-235 to 3199) | |  | 2675 (6064) | 4466 (8959) | 1792 (76 to 3508) | -310 (-2713 to 2094) | 0.80 |
| **eTable 1 Effects of Intervention on Primary and Secondary Outcomes (Full Results, Continued)** | | | | | | | | | | | |
| **Follow-up time** | **Run4Love Intervention Group (*N*=150)** | | | |  | | **Usual Care Group (*N*=150)** | | | **Between-Group Difference for Mean Change From Baseline (95% *CI*)** | ***P* value** |
|  | **Baseline, Mean (*SD*)** | **Follow-up, Mean (*SD*)** | **Within-Group Changes, Mean (95% *CI*)** ^c^ | |  | | **Baseline, Mean (*SD*)** | **Follow-up, Mean (*SD*)** | **Within-Group Changes, Mean (95% *CI*)** ^c^ |  |  |
| Self-efficacy GSES^b^ |  |  |  | |  | |  |  |  |  |  |
| 3-mon-follow-up | 24.4 (5.2) | 26.6 (6) | 2.24 (1.33 to 3.15) | |  | | 23.3 (5.6) | 23.4 (5.8) | 0.08 (-0.77 to 0.94) | 2.16 (0.92 to 3.40) | <0.001 |
| 6-mon-follow-up | 24.4 (5.2) | 26.5 (6) | 2.06 (1.09 to 3.03) | |  | | 23.3 (5.6) | 23.5 (5.7) | 0.20 (-0.76 to 1.15) | 1.86 (0.50 to 3.22) | 0.007 |
| 9-mon-follow-up | 24.4 (5.2) | 26.4 (5.9) | 2.01 (1.08 to 2.95) | |  | | 23.3 (5.6) | 23.8 (5.9) | 0.46 (-0.55 to 1.47) | 1.55 (0.19 to 2.91) | 0.025 |
| HIV Stigma Scale^a^ |  |  |  | |  | |  |  |  |  |  |
| 3-mon-follow-up | 37.1 (7.7) | 34.3 (9.1) | -2.85 (-4.09 to -1.61) | |  | | 38.0 (7.5) | 37.4 (8.3) | -0.56 (-1.65 to 0.53) | -2.29 (-3.93 to -0.65) | 0.006 |
| 6-mon-follow-up | 37.1 (7.7) | 34.2 (8.5) | -2.88 (-4.10 to -1.66) | |  | | 38.0 (7.5) | 37.2 (9.8) | -0.82 (-2.14 to 0.49) | -2.05 (-3.83 to -0.28) | 0.02 |
| 9-mon-follow-up | 37.1 (7.7) | 34.0 (9.0) | -3.15 (-4.43 to -1.86) | |  | | 38.0 (7.5) | 37.7 (9.9) | -0.28 (-1.60 to 1.04) | -2.87 (-4.71 to -1.03) | 0.002 |
| Depression severity PHQ-9^a^ |  |  |  | |  | |  |  |  |  |  |
| 3-mon-follow-up | 10.2 (4.5) | 6.8 (4.1) | -3.38 (-4.13 to -2.62) | |  | | 10.7 (5.1) | 8.9 (4.7) | -1.81 (-2.58 to -1.05) | -1.56 (-2.63 to -0.50) | 0.004 |
| 6-mon-follow-up | 10.2 (4.5) | 8.5 (4.9) | -1.68 (-2.54 to -0.81) | |  | | 10.7 (5.1) | 11.1 (5.5) | 0.34 (-0.49 to 1.16) | -2.01 (-3.20 to -0.83) | <0.001 |
| 9-mon-follow-up | 10.2 (4.5) | 9.1 (5.1) | -1.01 (-1.99 to -0.03) | |  | | 10.7 (5.1) | 10.9 (5.4) | 0.16 (-0.70 to 1.02) | -1.17 (-2.46 to 0.13) | 0.08 |
| Hair cortisol, pg/mg^a^ | 14.1 (21.9) | 22.9 (34.7) | 8.79 (2.30 to 15.27) | |  | | 17.4 (30.1) | 26.3 (42.2) | 8.81 (0.15 to 17.47) | -0.02 (-10.81 to 10.77) | 0.99 |
| Abbreviations: SD, standard deviation; IQR, interquartile range; CI, confidence interval; QOL, Quality of life; METs, Metabolic equivalents; CES-D, the Center for Epidemiological Studies Depression Scale (higher scores indicate greater depression); PHQ-9, 9-item Patient Health Questionnaire (higher scores indicate greater depression).  ^a^ A higher score indicates a worse outcome.  ^b^ A higher score indicates a better outcome.  ^c^ Within-group changes are mean changes. | | | | | | | | | | | |

| **eTable 2 Effects of Intervention on Primary and Secondary Outcomes before Missing Data Imputation** | | | | | | | | | | | |
| --- | --- | --- | --- | --- | --- | --- | --- | --- | --- | --- | --- |
| **Follow-up time** | **Run4Love Intervention Group** | | | |  | **Usual Care Group** | | | | **Between-Group Difference for Mean Change From Baseline (95% *CI*)** | ***P* value** |
|  | ***N*** | **Baseline, Mean (*SD*)** | **Follow-up, Mean (*SD*)** | **Within-Group Changes, Mean (95% *CI*)** ^c^ |  | **N** | **Baseline, Mean (*SD*)** | **Follow-up, Mean (*SD*)** | **Within-Group Changes, Mean (95% *CI*)** ^c^ |  |  |
| CES-D^a^ |  |  |  |  |  |  |  |  |  |  |  |
| 3-mon-follow-up | 139 | 24.2 (6.4) | 17.9 (9.4) | -6.29 (-7.76 to -4.83) |  | 135 | 24.1 (6.8) | 23.9 (10.1) | -0.20 (-1.71 to 1.31) | -6.09 (-8.19 to -4.00) | <0.001 |
| 6-mon-follow-up | 132 | 24.2 (6.5) | 17.6 (10.1) | -6.64 (-8.25 to -5.02) |  | 133 | 24.1 (6.7) | 24.1 (11.4) | -0.02 (-1.70 to 1.67) | -6.62 (-8.94 to -4.30) | <0.001 |
| 9-mon-follow-up | 133 | 23.9 (6.3) | 17.9 (10.7) | -6.06 (-7.91 to -4.21) |  | 127 | 24.4 (7.0) | 23.4 (11.5) | -0.93 (-2.67 to 0.81) | -5.13 (-7.66 to -2.60) | <0.001 |
| QOL^b^ |  |  |  |  |  |  |  |  |  |  |  |
| 3-mon-follow-up | 139 | 77.3 (9.2) | 82.5 (12.0) | 5.23 (3.62 to 6.83) |  | 135 | 76.3 (9.0) | 76.6 (11.1) | 0.37 (-0.95 to 1.68) | 4.86 (2.79 to 6.93) | <0.001 |
| 6-mon-follow-up | 132 | 77.3 (9.2) | 83.5 (12.9) | 6.19 (4.43 to 7.94) |  | 133 | 76.7 (9.6) | 76.3 (13.0) | -0.39 (-1.93 to 1.16) | 6.57 (4.24 to 8.90) | <0.001 |
| 9-mon-follow-up | 133 | 77.6 (9.3) | 83.5 (13.2) | 5.87 (3.78 to 7.96) |  | 127 | 76.1 (9.7) | 76.5 (13.3) | 0.49 (-1.28 to 2.26) | 5.38 (2.65 to 8.11) | <0.001 |
| Perceived stress PSS^a^ |  |  |  |  |  |  |  |  |  |  |  |
| 3-mon-follow-up | 139 | 20.0 (4.5) | 15.7 (5.8) | -4.32 (-5.18 to -3.47) |  | 135 | 21.0 (4.3) | 19.0 (5.4) | -1.93 (-2.76 to -1.1) | -2.39 (-3.58 to -1.20) | <0.001 |
| 6-mon-follow-up | 132 | 20.0 (4.6) | 16.4 (5.9) | -3.52 (-4.44 to -2.59) |  | 133 | 20.9 (4.4) | 19.4 (6.1) | -1.53 (-2.4 to -0.65) | -1.99 (-3.26 to -0.72) | 0.002 |
| 9-mon-follow-up | 133 | 20.1 (4.6) | 16.1 (6.0) | -3.99 (-4.91 to -3.07) |  | 127 | 20.9 (4.3) | 18.7 (6.0) | -2.22 (-3.15 to -1.29) | -1.77 (-3.08 to -0.47) | 0.008 |
| SWCQ positive coping^b^ |  |  |  |  |  |  |  |  |  |  |  |
| 3-mon-follow-up | 139 | 18.4 (5.6) | 20.8 (7.3) | 2.42 (1.23 to 3.62) |  | 135 | 18.3 (5.9) | 17.7 (5.9) | -0.62 (-1.55 to 0.30) | 3.05 (1.54 to 4.55) | <0.001 |
| 6-mon-follow-up | 132 | 18.6 (5.6) | 21.0 (7.5) | 2.45 (1.25 to 3.66) |  | 133 | 18.5 (6.1) | 17.4 (6.6) | -1.13 (-2.22 to -0.03) | 3.58 (1.96 to 5.20) | <0.001 |
| 9-mon-follow-up | 133 | 18.5 (5.6) | 21.0 (7.8) | 2.43 (1.12 to 3.74) |  | 127 | 18.5 (6.2) | 18.3 (6.4) | -0.22 (-1.36 to 0.92) | 2.65 (0.92 to 4.38) | 0.003 |
| SWCQ negative coping^b^ |  |  |  |  |  |  |  |  |  |  |  |
| 3-mon-follow-up | 139 | 11.8 (3.8) | 11.1 (4.3) | -0.68 (-1.43 to 0.06) |  | 135 | 11.8 (3.9) | 11.4 (3.7) | -0.39 (-1.03 to 0.24) | -0.29 (-1.26 to 0.68) | 0.56 |
| 6-mon-follow-up | 132 | 11.9 (3.9) | 11.3 (4.4) | -0.55 (-1.27 to 0.18) |  | 133 | 11.7 (3.9) | 11.3 (4.1) | -0.38 (-1.08 to 0.33) | -0.17 (-1.17 to 0.83) | 0.74 |
| 9-mon-follow-up | 133 | 11.9 (3.9) | 11.7 (4.1) | -0.15 (-0.91 to 0.61) |  | 127 | 11.9 (4.0) | 11.9 (4.1) | -0.02 (-0.83 to 0.79) | -0.13 (-1.23 to 0.98) | 0.82 |
| Physical activity METs^b^ |  |  |  |  |  |  |  |  |  |  |  |
| 3-mon-follow-up | 139 | 3323.3 (6346.1) | 3052.4 (5822.8) | -270.9 (-1378.9 to 837.1) |  | 135 | 2855.4 (6354.5) | 4545.0 (16437.6) | 1689.6 (-579.7 to 3959.0) | -1960.5 (-4479.0 to 557.8) | 0.13 |
| 6-mon-follow-up | 132 | 3386.2 (6465.5) | 4405.6 (12003.9) | 1019.4 (-1019.0 to 3057.8) |  | 132 | 2870.6 (6408.1) | 3986.3 (9040.0) | 1115.7 (-768.6 to 3000.1) | -96.3 (-2859.5 to 2666.8) | 0.95 |
| 9-mon-follow-up | 132 | 3409.94(6456.0) | 4734.5 (10363.3) | 1324.5 (-472.5 to 3121.5) |  | 127 | 2896.7 (6530.8) | 4602.8 (9243.3) | 1706.14 (-164.4 to 3576.7) | -381.6 (-2963.2 to 2200.0) | 0.77 |

| **eTable 2 Effects of Intervention on Primary and Secondary Outcomes before Missing Data Imputation (Continued)** | | | | | | | | | | | |
| --- | --- | --- | --- | --- | --- | --- | --- | --- | --- | --- | --- |
| **Follow-up time** | **Run4Love Intervention Group** | | | |  | **Usual Care Group** | | | | **Between-Group Difference for Mean Change From Baseline (95% *CI*)** | ***P* value** |
|  | ***N*** | **Baseline, Mean (*SD*)** | **Follow-up, Mean (*SD*)** | **Within-Group Changes, Mean (95% *CI*)** ^c^ |  | ***N*** | **Baseline, Mean (*SD*)** | **Follow-up, Mean (*SD*)** | **Within-Group Changes, Mean (95% *CI*)** ^c^ |  |  |
| Self-efficacy GSES^b^ |  |  |  |  |  |  |  |  |  |  |  |
| 3-mon-follow-up | 139 | 24.3 (5.2) | 26.6 (6.0) | 2.32 (1.41 to 3.24) |  | 135 | 23.2 (5.6) | 23.4 (5.8) | 0.18 (-0.68 to 1.03) | 2.15 (0.90 to 3.39) | <0.001 |
| 6-mon-follow-up | 132 | 24.3 (5.3) | 26.4 (6.0) | 2.09 (1.10 to 3.08) |  | 133 | 23.4 (5.6) | 23.7 (5.7) | 0.29 (-0.67 to 1.26) | 1.80 (0.42 to 3.17) | 0.011 |
| 9-mon-follow-up | 133 | 24.4 (5.3) | 26.6 (5.8) | 2.18 (1.27 to 3.09) |  | 127 | 23.4 (5.7) | 23.8 (5.9) | 0.39 (-0.68 to 1.47) | 1.79 (0.38 to 3.19) | 0.013 |
| HIV Stigma Scale^a^ |  |  |  |  |  |  |  |  |  |  |  |
| 3-mon-follow-up | 139 | 37.3 (7.7) | 34.3 (9.2) | -3.04 (-4.29 to -1.78) |  | 135 | 38.0 (7.5) | 37.5 (8.3) | -0.50 (-1.59 to 0.58) | -2.53 (-4.19 to -0.88) | 0.003 |
| 6-mon-follow-up | 132 | 37.4 (7.9) | 34.3 (8.5) | -3.11 (-4.35 to -1.87) |  | 133 | 38.2 (7.5) | 37.4 (9.9) | -0.83 (-2.20 to 0.53) | -2.27 (-4.11 to -0.44) | 0.015 |
| 9-mon-follow-up | 133 | 37.0 (8.0) | 34.0 (9.0) | -3.05 (-4.36 to -1.75) |  | 127 | 38.1 (7.8) | 37.8 (10.0) | -0.27 (-1.61 to 1.08) | -2.78 (-4.65 to -0.92) | 0.004 |
| Depression severity PHQ-9^a^ |  |  |  |  |  |  |  |  |  |  |  |
| 3-mon-follow-up | 139 | 10.2 (4.6) | 6.8 (4.1) | -3.43 (-4.19 to -2.68) |  | 135 | 10.8 (5.0) | 9.0 (4.6) | -1.81 (-2.59 to -1.02) | -1.62 (-2.71 to -0.54) | 0.003 |
| 6-mon-follow-up | 132 | 10.3 (4.6) | 8.5 (5.0) | -1.80 (-2.68 to -0.92) |  | 133 | 10.8 (5.2) | 11.2 (5.6) | 0.40 (-0.44 to 1.24) | -2.19 (-3.40 to -0.98) | <0.001 |
| 9-mon-follow-up | 133 | 10.2 (4.7) | 9.2 (5.1) | -1.06 (-2.09 to -0.03) |  | 127 | 10.8 (5.2) | 10.9 (5.5) | 0.10 (-0.78 to 0.98) | -1.16 (-2.51 to 0.19) | 0.09 |
| Hair cortisol to pg/mg^a^ | 135 | 14.46 (22.83) | 23.05 (35.38) | 8.59 (1.71 to 15.48) |  | 129 | 17.03 (29.3) | 26.43 (42.4) | 9.40 (0.63 to 18.16) | -0.80 (-11.90 to 10.3) | 0.89 |
| Abbreviations: SD, standard deviation; IQR, interquartile range; CI, confidence interval; QOL, Quality of life; METs, Metabolic equivalents; CES-D, the Center for Epidemiological Studies Depression Scale (higher scores indicate greater depression); PHQ-9, 9-item Patient Health Questionnaire (higher scores indicate greater depression).  ^a^ A higher score indicates a worse outcome.  ^b^ A higher score indicates a better outcome.  ^c^ Within-group changes are mean changes. | | | | | | | | | | | |

| **eTable 3 Linear Mixed-Effects Model Results of the Primary Outcome and Secondary Outcomes^a^** | | | | |
| --- | --- | --- | --- | --- |
| **Variables** | **Beta coefficient** | **Standard error** | **95% *CI*** | ***P* value** |
| CES-D |  |  |  |  |
| Intercept | 24.69 | 5.03 | 14.84 to 34.54 | <0.001 |
| Group | -0.14 | 0.78 | -1.67 to 1.39 | 0.85 |
| 3-month vs BL | -0.37 | 0.74 | -1.82 to 1.09 | 0.62 |
| 6-month vs BL | -0.25 | 0.81 | -1.84 to 1.35 | 0.76 |
| 9-month vs BL | -0.74 | 0.89 | -2.48 to 0.99 | 0.40 |
| Group×(3-month vs BL) | -5.80 | 1.05 | -7.86 to -3.74 | <0.001 |
| Group×(6-month vs BL) | -6.18 | 1.15 | -8.44 to -3.92 | <0.001 |
| Group×(9-month vs BL) | -5.46 | 1.25 | -7.91 to -3.02 | <0.001 |
| QOL |  |  |  |  |
| Intercept | 68.59 | 6.88 | 55.1 to 82.07 | <0.001 |
| Group | 0.62 | 1.06 | -1.46 to 2.70 | 0.56 |
| 3-month vs BL | 0.32 | 0.74 | -1.13 to 1.78 | 0.66 |
| 6-month vs BL | -0.28 | 0.83 | -1.90 to 1.34 | 0.73 |
| 9-month vs BL | 0.05 | 0.96 | -1.82 to 1.92 | 0.96 |
| Group×(3-month vs BL) | 4.81 | 1.04 | 2.76 to 6.85 | <0.001 |
| Group×(6-month vs BL) | 6.51 | 1.17 | 4.21 to 8.81 | <0.001 |
| Group×(9-month vs BL) | 5.85 | 1.35 | 3.21 to 8.49 | <0.001 |
| Depression severity PHQ-9 |  |  |  |  |
| Intercept | 15.02 | 3.10 | 8.94 to 21.09 | <0.001 |
| Group | -0.55 | 0.56 | -1.65 to 0.55 | 0.33 |
| 3-month vs BL | -1.80 | 0.38 | -2.55 to -1.05 | <0.001 |
| 6-month vs BL | 0.37 | 0.42 | -0.46 to 1.20 | 0.38 |
| 9-month vs BL | 0.13 | 0.47 | -0.78 to 1.05 | 0.78 |
| Group×(3-month vs BL) | -1.60 | 0.54 | -2.65 to -0.54 | 0.003 |
| Group×(6-month vs BL) | -2.07 | 0.60 | -3.25 to -0.90 | <0.001 |
| Group×(9-month vs BL) | -1.19 | 0.66 | -2.47 to 0.10 | 0.07 |
| Self-efficacy GSES |  |  |  |  |
| Intercept | 23.28 | 3.64 | 16.14 to 30.41 | <0.001 |
| Group | 1.07 | 0.63 | -0.16 to 2.30 | 0.09 |
| 3-month vs BL | 0.09 | 0.45 | -0.78 to 0.96 | 0.84 |
| 6-month vs BL | 0.18 | 0.48 | -0.77 to 1.13 | 0.71 |
| 9-month vs BL | 0.42 | 0.50 | -0.55 to 1.40 | 0.39 |
| Group×(3-month vs BL) | 2.17 | 0.63 | 0.95 to 3.40 | <0.001 |
| Group×(6-month vs BL) | 1.90 | 0.68 | 0.56 to 3.24 | 0.006 |
| Group×(9-month vs BL) | 1.62 | 0.70 | 0.25 to 2.99 | 0.02 |

| **eTable 3 Linear Mixed-Effects Model Results of the Primary Outcome and Secondary Outcomes^a^ (Continued)** | | | | |
| --- | --- | --- | --- | --- |
| **Variables** | **Beta coefficient** | **Standard error** | **95% *CI*** | ***P* value** |
| Perceived stress PSS |  |  |  |  |
| Intercept | 22.75 | 3.21 | 16.46 to 29.03 | <0.001 |
| Group | -0.75 | 0.51 | -1.75 to 0.25 | 0.14 |
| 3-month vs BL | -1.81 | 0.42 | -2.64 to -0.98 | <0.001 |
| 6-month vs BL | -1.46 | 0.45 | -2.33 to -0.59 | 0.001 |
| 9-month vs BL | -2.07 | 0.46 | -2.97 to -1.17 | <0.001 |
| Group×(3-month vs BL) | -2.46 | 0.60 | -3.63 to -1.29 | <0.001 |
| Group×(6-month vs BL) | -1.92 | 0.63 | -3.16 to -0.68 | 0.002 |
| Group×(9-month vs BL) | -1.79 | 0.65 | -3.06 to -0.52 | 0.006 |
| HIV Stigma Scale |  |  |  |  |
| Intercept | 38.47 | 5.72 | 27.26 to 49.67 | <0.001 |
| Group | -0.77 | 0.90 | -2.54 to 0.99 | 0.39 |
| 3-month vs BL | -0.60 | 0.59 | -1.76 to 0.56 | 0.31 |
| 6-month vs BL | -0.80 | 0.64 | -2.06 to 0.46 | 0.21 |
| 9-month vs BL | -0.26 | 0.66 | -1.55 to 1.03 | 0.69 |
| Group×(3-month vs BL) | -2.30 | 0.83 | -3.93 to -0.66 | 0.006 |
| Group×(6-month vs BL) | -2.20 | 0.91 | -3.99 to -0.41 | 0.02 |
| Group×(9-month vs BL) | -2.89 | 0.93 | -4.71 to -1.06 | 0.002 |
| Depression severity PHQ-9 |  |  |  |  |
| Intercept | 15.02 | 3.10 | 8.94 to 21.09 | <0.001 |
| Group | -1.80 | 0.38 | -2.55 to -1.05 | <0.001 |
| 3-month vs BL | 0.37 | 0.42 | -0.46 to 1.20 | 0.38 |
| 6-month vs BL | 0.13 | 0.47 | -0.78 to 1.05 | 0.78 |
| 9-month vs BL | -0.55 | 0.56 | -1.65 to 0.55 | 0.33 |
| Group×(3-month vs BL) | -1.60 | 0.54 | -2.65 to -0.54 | 0.003 |
| Group×(6-month vs BL) | -2.07 | 0.60 | -3.25 to -0.90 | <0.001 |
| Group×(9-month vs BL) | -1.19 | 0.66 | -2.47 to 0.10 | 0.07 |
| SWCQ positive coping |  |  |  |  |
| Intercept | 17.71 | 3.97 | 9.94 to 25.49 | <0.001 |
| Group | -0.27 | 0.67 | -1.58 to 1.05 | 0.69 |
| 3-month vs BL | -0.59 | 0.54 | -1.65 to 0.47 | 0.28 |
| 6-month vs BL | -0.99 | 0.57 | -2.12 to 0.13 | 0.08 |
| 9-month vs BL | -0.18 | 0.61 | -1.38 to 1.01 | 0.77 |
| Group×(3-month vs BL) | 2.95 | 0.76 | 1.46 to 4.44 | <0.001 |
| Group×(6-month vs BL) | 3.49 | 0.81 | 1.89 to 5.08 | <0.001 |
| Group×(9-month vs BL) | 2.61 | 0.86 | 0.93 to 4.29 | 0.002 |

| **eTable 3 Linear Mixed-Effects Model Results of the Primary Outcome and Secondary Outcomes^a^ (Continued)** | | | | |
| --- | --- | --- | --- | --- |
| **Variables** | **Beta coefficient** | **Standard error** | **95% *CI*** | ***P* value** |
| SWCQ negative coping |  |  |  |  |
| Intercept | 10.86 | 2.46 | 6.04 to 15.67 | <0.001 |
| Group | -0.05 | 0.44 | -0.92 to 0.82 | 0.91 |
| 3-month vs BL | -0.34 | 0.35 | -1.02 to 0.34 | 0.33 |
| 6-month vs BL | -0.43 | 0.35 | -1.13 to 0.26 | 0.22 |
| 9-month vs BL | 0.01 | 0.38 | -0.74 to 0.76 | 0.98 |
| Group×(3-month vs BL) | -0.34 | 0.49 | -1.30 to 0.62 | 0.49 |
| Group×(6-month vs BL) | -0.004 | 0.50 | -0.99 to 0.98 | 0.99 |
| Group×(9-month vs BL) | -0.11 | 0.54 | -1.17 to 0.95 | 0.84 |
| Abbreviations: CI, confidence interval; BL, Baseline; BMI, Body Mass Index; CES-D, the Center for Epidemiological Studies Depression Scale; PHQ-9, 9-item Patient Health Questionnaire; QOL, quality of life; GSES, General Self-efficacy Scale; PSS, Perceived Stress Scale; SWCQ, Simplified Ways of Coping Questionnaire.  ^a^ Adjusted for age, gender, BMI, education, sexual orientation, family monthly income, marital status, duration of HIV infection, and employment.  Explanation of the model: Intercept, mean value in usual care group at baseline; Group, between-group difference at baseline; 3-, 6-, 9-month vs BL, difference from baseline in usual care group at 3, 6, or 9 months; Group×(3-, 6-, 9-month vs BL), interaction effect, representing between-group difference for mean change from baseline. | | | | |

| **eTable 4 Effects of Intervention on Depression severity PHQ-9 ≥ 10** | | | | |
| --- | --- | --- | --- | --- |
| **Follow-up time** | **Run4Love Intervention Group (*N*=150), No. (%) [95% *CI*]** | **Usual Care Group (*N*=150), No. (%) [95% *CI*]** | **Between-Group Difference in Percentage Points (95% *CI*)** | ***P* value^a^** |
| Depression severity PHQ-9 ≥10 |  |  |  |  |
| 3-mon-follow-up | 32 (21.3) [14.8 to 27.9] | 62 (41.3) [33.5 to 49.2] | -20.0 (-30.1 to -9.7) | <0.001 |
| 6-mon-follow-up | 55 (36.7) [29.0 to 44.4] | 92 (61.3) [53.5 to 69.1] | -24.7 (-35.6 to -13.7) | <0.001 |
| 9-mon-follow-up | 65 (43.3) [35.4 to 51.3] | 92 (61.3) [53.5 to 69.1] | -18.0 (-29.1 to -6.9) | 0.002 |
| Abbreviations: CI, confidence interval; PHQ-9, 9-item Patient Health Questionnaire (higher scores indicate greater depression).  ^a^ *H_0_*, the risk difference equals to zero. | | | | |

| **eTable 5 Generalized Estimating Equation Analysis of Run4Love Effects on Percentage of PHQ-9 ≥ 10 ^a^** | | | | |
| --- | --- | --- | --- | --- |
| **Variables** | **Beta coefficient** | ***RR*** | **95% *CI*** | ***P* value** |
| Intercept | 1.52 | 4.56 | 0.43 to 48.02 | 0.21 |
| Group | 0.03 | 1.03 | 0.65 to 1.64 | 0.89 |
| 3-month vs BL | -0.35 | 0.71 | 0.48 to 1.04 | 0.08 |
| 6-month vs BL | 0.42 | 1.53 | 1.08 to 2.16 | 0.02 |
| 9-month vs BL | 0.44 | 1.55 | 1.06 to 2.25 | 0.02 |
| Group×(3-month vs BL) | -1.10 | 0.33 | 0.18 to 0.62 | <0.001 |
| Group×(6-month vs BL) | -1.11 | 0.33 | 0.19 to 0.57 | <0.001 |
| Group×(9-month vs BL) | -0.86 | 0.42 | 0.24 to 0.74 | 0.003 |
| Abbreviations: RR, risk ratio; CI, confidence interval; BL, Baseline; PHQ-9, 9-item Patient Health Questionnaire.  ^a^ Adjusted for age, gender, BMI, education, sexual orientation, family monthly income, marital status, duration of HIV infection, and employment.  Explanation of the model: The model shows that the Run4Love intervention reduced the risk of moderate to severe PHQ-9 depression (PHQ-9 ≥ 10) at the 3-, 6-, and 9-month assessments compared with the usual care group. | | | | |

| **eFigure 1. Secondary Outcomes Over Time for the Run4Love Intervention vs Control Groups** |
| --- |
| 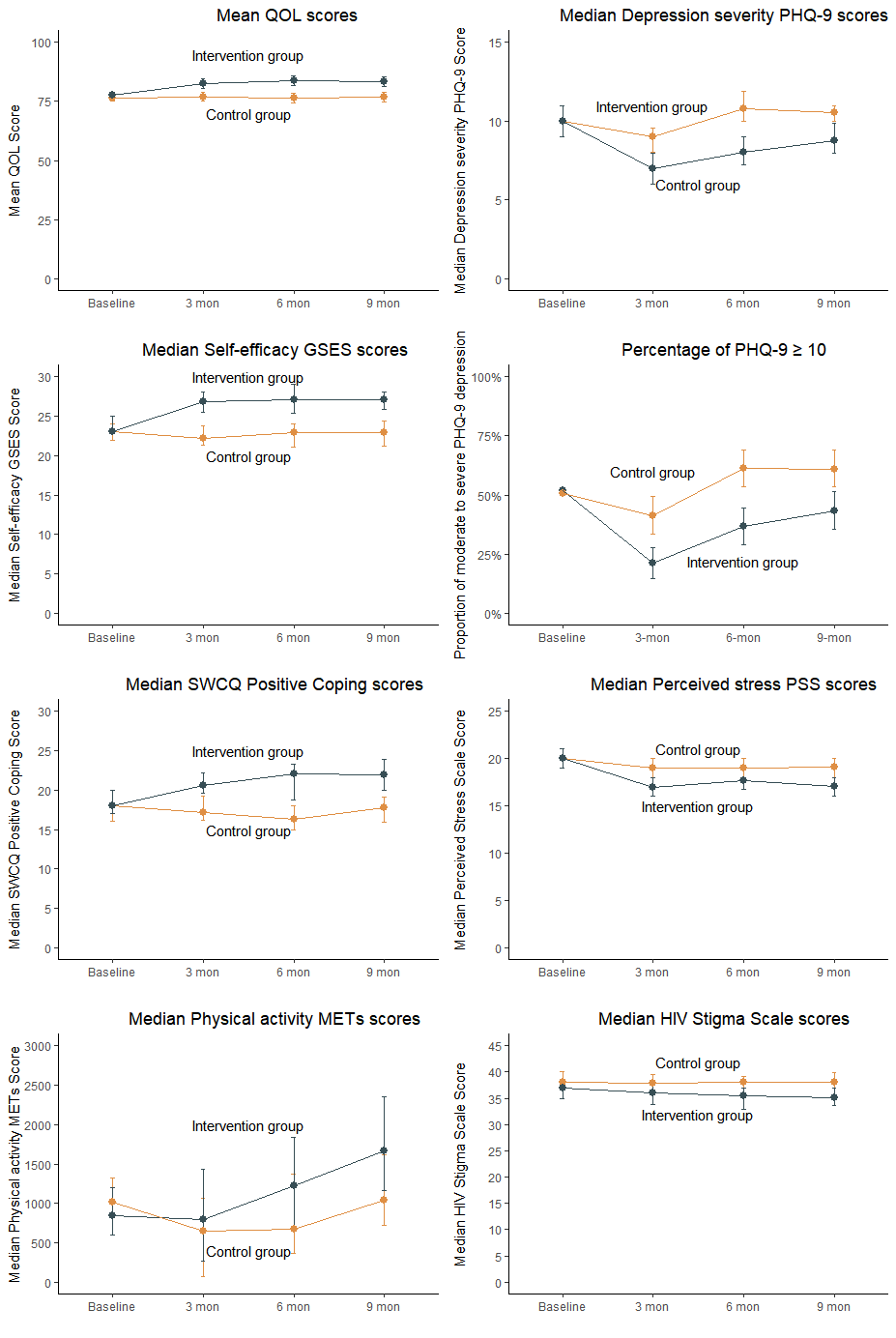 |
| Abbreviations: PHQ-9, 9-item Patient Health Questionnaire; METs, Metabolic equivalents; QOL, quality of life; GSES, General Self-efficacy Scale; PSS, Perceived Stress Scale; SWCQ, Simplified Ways of Coping Questionnaire.  Error bars indicate 95% CIs. |

| **eTable 6 Comparison of Participants between Who Were Lost Before 3-month Evaluation and Completed 3-month Outcome Evaluation** | | | |
| --- | --- | --- | --- |
| **Variables** | **Lost before 3-month evaluation (*N*=26)** | **Completed 3-month outcome evaluation (*N*=274)** | ***P* value** |
| Age, median (IQR) | 30.1 (27.6 to 35.9) | 27.2 (24.1 to 31.2) | 0.001 |
| Male, No. (%) | 24 (92.3) | 253 (92.3) | 0.53 |
| BMI, median (IQR) | 20.6 (19.0 to 21.5) | 19.8 (18.5 to 21.3) | 0.50 |
| Education level>high school, No. (%) | 14 (53.9) | 168 (61.3) | 0.53 |
| Homosexual/bisexual/uncertain, No. (%) | 19 (73.1) | 226 (82.5) | 0.29 |
| Married, No. (%) | 3 (11.5) | 35 (12.8) | >0.99 |
| Family monthly income ≥ 7000, No. (%) | 15 (57.7) | 109 (39.8) | 0.10 |
| Duration of HIV infection, median (IQR) | 2.05 (0.56 to 4.83) | 1.71 (0.59 to 3.66) | 0.47 |
| CES-D, median (IQR) | 22.0 (19.0 to 25.8) | 23.0 (19.0 to 28.0) | 0.67 |
| Depression severity PHQ-9, median (IQR) | 8.00 (7.00 to 12.00) | 10.00 (7.00 to 13.00) | 0.19 |
| Physical activity METs, median (IQR) | 744 (0 to 1950) | 880 (160 to 2490) | 0.17 |
| QOL, mean (SD) | 79.3 (10.4) | 76.8 (9.1) | 0.25 |
| Self-efficacy GSES, median (IQR) | 25.0 (20.3 to 27.8) | 23.0 (20.0 to 27.0) | 0.38 |
| Perceived stress PSS, median (IQR) | 19.0 (17.0 to 20.8) | 20.0 (18.0 to 23.0) | 0.05 |
| HIV Stigma Scale, median (IQR) | 37.5 (31.3 to 40.0) | 38.0 (32.0 to 42.0) | 0.34 |
| SWCQ positive coping, median (IQR) | 18.5 (14.0 to 22.5) | 18.0 (14.0 to 22.0) | 0.91 |
| SWCQ negative coping, median (IQR) | 11.0 (8.3 to 14.8) | 12.0 (9.0 to 15.0) | 0.49 |
| Abbreviations: SD, standard deviation; IQR, interquartile range; BMI, Body Mass Index; CES-D, the Center for Epidemiological Studies Depression Scale; PHQ-9, 9-item Patient Health Questionnaire; METs, Metabolic equivalents; QOL, quality of life; GSES, General Self-efficacy Scale; PSS, Perceived Stress Scale; SWCQ, Simplified Ways of Coping Questionnaire. | | | |

| **eTable 7 Comparison of Participants between Who Were Lost Before 6-month Evaluation and Completed 6-month Outcome Evaluation** | | | |
| --- | --- | --- | --- |
| **Variables** | **Lost before 6-month evaluation (*N*=35)** | **Completed 6-month outcome evaluation (*N*=265)** | ***P* value** |
| Age, median (IQR) | 30.3 (25.9 to 35.2) | 27.3 (24.0 to 31.0) | 0.002 |
| Male, No. (%) | 30 (85.7) | 247 (93.2) | 0.17 |
| BMI, median (IQR) | 20.6 (19.1 to 22.4) | 20.1 (18.6 to 21.7) | 0.33 |
| Education level>high school, No. (%) | 22 (62.9) | 160 (60.4) | 0.86 |
| Homosexual/bisexual/uncertain, No. (%) | 28 (80.0) | 217 (81.9) | 0.82 |
| Married, No. (%) | 4 (11.4) | 34 (12.8) | >0.99 |
| Family monthly income ≥ 7000, No. (%) | 18 (51.4) | 106 (40.0) | 0.21 |
| Duration of HIV infection, median (IQR) | 1.90 (0.33 to 5.00) | 1.72 (0.60 to 3.65) | 0.51 |
| CES-D, median (IQR) | 22.0 (19.0, 26.0) | 23.0 (19.0, 28.0) | 0.35 |
| Depression severity PHQ-9, median (IQR) | 9.0 (7.0., 12.0) | 10.0 (7.0, 13.0) | 0.48 |
| Physical activity METs, median (IQR) | 600 (0, 2264) | 960 (200, 2400) | 0.08 |
| QOL, mean (SD) | 77.0 ± 8.0 | 77.0 ± 9.4 | 0.99 |
| Self-efficacy GSES, median (IQR) | 22.0 (20.0, 27.0) | 23.0 (20.0, 28.0) | 0.74 |
| Perceived stress PSS, median (IQR) | 20.0 (18.5, 22.0) | 20.0 (18.0, 23.0) | 0.77 |
| HIV Stigma Scale, median (IQR) | 36.0 (32.0, 40.0) | 38.0 (32.0, 42.0) | 0.10 |
| SWCQ positive coping, median (IQR) | 16.0 (14.0, 20.0) | 18.0 (14.0, 22.0) | 0.12 |
| SWCQ negative coping, median (IQR) | 12.0 (8.0, 15.0) | 12.0 (9.0, 15.0) | 0.90 |
| Abbreviations: SD, standard deviation; IQR, interquartile range; BMI, Body Mass Index; CES-D, the Center for Epidemiological Studies Depression Scale; PHQ-9, 9-item Patient Health Questionnaire; METs, Metabolic equivalents; QOL, quality of life; GSES, General Self-efficacy Scale; PSS, Perceived Stress Scale; SWCQ, Simplified Ways of Coping Questionnaire. | | | |

| **eTable 8 Comparison of Participants between Who Were Lost Before 9-month Evaluation and Completed 9-month Outcome Evaluation** | | | |
| --- | --- | --- | --- |
| **Variables** | **Lost before 9-month evaluation (*N*=40)** | **Completed 9-month outcome evaluation (*N*=260)** | ***P* value** |
| Age, median (IQR) | 28.9 (25.3 to 34.0) | 27.4 (24.0 to 31.1) | 0.04 |
| Male, No. (%) | 36 (90.0) | 241 (92.7) | 0.53 |
| BMI, median (IQR) | 20.6 (19.1 to 22.5) | 20.0 (18.6 to 21.6) | 0.17 |
| Education level>high school, No. (%) | 24 (60.0) | 158 (60.8) | >0.99 |
| Homosexual/bisexual/uncertain, No. (%) | 33 (82.5) | 212 (81.5) | >0.99 |
| Married, No. (%) | 4 (10.0) | 34 (13.1) | 0.80 |
| Family monthly income ≥ 7000, No. (%) | 17 (42.5) | 107 (41.2) | 0.87 |
| Duration of HIV infection, median (IQR) | 2.00 (0.90 to 4.00) | 1.70 (0.60 to 3.70) | 0.39 |
| CES-D, median (IQR) | 22.0 (19.0, 27.0) | 23.0 (19.0, 28.0) | 0.80 |
| Depression severity PHQ-9, median (IQR) | 9.0 (7.0, 12.0) | 10.0 (7.0, 13.0) | 0.51 |
| Physical activity METs, median (IQR) | 520 (0, 2430) | 960 (190, 2400) | 0.12 |
| QOL, mean (SD) | 78.1 ± 7.3 | 76.9 ± 9.5 | 0.36 |
| Self-efficacy GSES, median (IQR) | 22.0 (20.0, 27.3) | 23.0 (20.0, 28.0) | 0.41 |
| Perceived stress PSS, median (IQR) | 20.0 (18.0, 21.0) | 20.0 (18.0, 23.0) | 0.44 |
| HIV Stigma Scale, median (IQR) | 38.0 (34.5, 40.3) | 37.5 (32.0, 42.0) | 0.89 |
| SWCQ positive coping, median (IQR) | 16.0 (13.8, 21.0) | 18.5 (14.0, 22.0) | 0.16 |
| SWCQ negative coping, median (IQR) | 11.5 (8.0, 13.0) | 12.0 (9.0, 15.0) | 0.23 |
| Abbreviations: SD, standard deviation; IQR, interquartile range; BMI, Body Mass Index; CES-D, the Center for Epidemiological Studies Depression Scale; PHQ-9, 9-item Patient Health Questionnaire; METs, Metabolic equivalents; QOL, quality of life; GSES, General Self-efficacy Scale; PSS, Perceived Stress Scale; SWCQ, Simplified Ways of Coping Questionnaire. | | | |

| **eTable 9 Effect Size of Intervention on Primary and Secondary Outcomes** | | | |
| --- | --- | --- | --- |
| **Variables** | **3-month follow-up** | **6-month follow-up** | **9-month follow-up** |
| CES-D | 0.66 | 0.63 | 0.51 |
| Perceived stress PSS | 0.49 | 0.36 | 0.34 |
| SWCQ positive coping | 0.45 | 0.51 | 0.36 |
| SWCQ negative coping | 0.09 | <0.01 | 0.02 |
| Physical activity METs | 0.19 | 0.01 | 0.03 |
| Self-efficacy GSES | 0.41 | 0.33 | 0.27 |
| QOL | 0.55 | 0.68 | 0.52 |
| HIV Stigma Scale | 0.33 | 0.27 | 0.37 |
| Depression severity PHQ-9 | 0.34 | 0.40 | 0.21 |
| Abbreviations: CES-D, the Center for Epidemiological Studies Depression Scale; PHQ-9, 9-item Patient Health Questionnaire; METs, Metabolic equivalents; QOL, quality of life; GSES, General Self-efficacy Scale; PSS, Perceived Stress Scale; SWCQ, Simplified Ways of Coping Questionnaire. | | | |

**eTable 10 Results of Patient Satisfaction between the Intervention and Control Groups**

|  | **Group** | |  |  |
| --- | --- | --- | --- | --- |
| **Satisfaction** | **Intervention *n* (%)** | **Control *n* (%)** | ***Chi-square*** | ***P* value** |
| Satisfied |  |  |  |  |
| 3-mon-follow-up | 131 (94.2) | 124 (91.9) | 0.608 | 0.436 |
| 6-mon-follow-up | 122 (92.4) | 126 (94.7) | 0.590 | 0.442 |
| 9-mon-follow-up | 123 (92.5) | 124 (97.6) | 3.637 | 0.057 |

3-mon-follow-up: intervention group *N*=139, control group *N*=135;

6-mon-follow-up: intervention group *N*=132, control group *N*=133;

9-mon-follow-up: intervention group *N*=133, control group *N*=127.
